# Kinetics of Hepatitis E Virus Infections in Asymptomatic Persons

**DOI:** 10.3201/eid3005.231764

**Published:** 2024-05

**Authors:** Ricarda Plümers, Jens Dreier, Cornelius Knabbe, Eike Steinmann, Daniel Todt, Tanja Vollmer

**Affiliations:** Herz- und Diabeteszentrum Nordrhein-Westfalen, Bad Oeynhausen, Germany (R. Plümers, J. Dreier, C. Knabbe, T. Vollmer);; German Centre for Infection Research, Bochum (E. Steinmann);; Ruhr University Bochum, Germany (E. Steinmann, D. Todt);; European Virus Bioinformatics Center, Jena, Germany (D. Todt)

**Keywords:** Hepatitis E virus, hepatitis, asymptomatic, doubling time, immune response, viruses, Germany, transfusion, blood safety

## Abstract

To determine the kinetics of hepatitis E virus (HEV) in asymptomatic persons and to evaluate viral load doubling time and half-life, we retrospectively tested samples retained from 32 HEV RNA-positive asymptomatic blood donors in Germany. Close-meshed monitoring of viral load and seroconversion in intervals of ≈4 days provided more information about the kinetics of asymptomatic HEV infections. We determined that a typical median infection began with PCR-detectable viremia at 36 days and a maximum viral load of 2.0 × 10^4^ IU/mL. Viremia doubled in 2.4 days and had a half-life of 1.6 days. HEV IgM started to rise on about day 33 and peaked on day 36; IgG started to rise on about day 32 and peaked on day 53**.** Although HEV IgG titers remained stable, IgM titers became undetectable in 40% of donors. Knowledge of the dynamics of HEV viremia is useful for assessing the risk for transfusion-transmitted hepatitis E.

Hepatitis E virus (HEV; family *Hepeviridae*), is a single-stranded positive-sense RNA virus with 3 open reading frames (*1*). Members of the HEV species *Paslahepevirus balayani* have been assigned to 8 genotypes, of which genotypes HEV-1 through HEV-4 are particularly relevant for human infection (*1*,*2*). Although infections with HEV-1 and HEV-2 are endemic to tropical countries and are transmitted by the oral–fecal route, HEV-3 and HEV-4 are transmitted zoonotically and infections are mainly found in Europe, North and South America, and Asia (*3*).

The dynamics of HEV infections are diverse among the cases described in the literature, which almost exclusively describe progression in symptomatic persons. Knowledge of progression of asymptomatic infection is limited, partly because asymptomatic infections are normally not detected. However, detection of HEV infection has increased in recent years, after analytical testing of blood donations for HEV became a focus of attention to improve the safety of blood transfusions. Systematic blood donor screening offers the possibility of identifying a large number of asymptomatic cases. Since 2004, transfusion-transmitted HEV infections have been repeatedly reported and pose a high risk for symptomatic or even chronic progression, particularly in immunosuppressed patients (*4*–*6*). 

HEV infections in otherwise healthy persons are usually asymptomatic and self-limiting (*7*). However, according to the World Health Organization, using estimates from 2015 data, HEV is a leading cause for acute viral hepatitis in 20 million persons annually, including 3.3 million with symptomatic cases and 44,000 HEV-related deaths (*8*). Collecting data on the progression of viral load over the course of acute infection is difficult. The onset of symptoms coincides with the peak of viremia, and the first phase of infection cannot be analyzed (*9*). In addition, a large proportion of infections are not investigated because of the absence of symptoms. If HEV infection is identified by chance, such as during screening as part of quality assurance of blood products after donation, the donor is suspended from donation (*10*). For that reason, the data on HEV antibody detection are denser, and data on viremia are lacking, especially for persons with asymptomatic infections.

Several factors can be used to consider the course of infection progression, including the maximum viral load and when it is reached, depending on the time of infection or onset of signs/symptoms. Furthermore, the time it takes for the viral load to double before reaching the maximum load and the half-life of the viral load after that point can provide information about the nature of the infection. Comparable data have been collected (e.g., for hepatitis A [HAV], B [HBV], and C [HCV] viruses) from healthy blood donors or symptomatic patients (*11*–*14*).

An incubation period of 2–9 weeks is assumed for HEV infections (*15*). HEV is detectable in blood samples for ≈4 weeks and in fecal samples for 6 weeks by screening with nucleic acid amplification testing (NAT) (*16*). Studies of the serologic status of HEV-infected rhesus monkeys and patients with acute hepatitis E have shown increased HEV IgG and IgM titers 3–4 weeks after HEV RNA detection-based confirmation of infection. Although HEV IgM is detectable for only a few months, HEV IgG remains for years (*17*–*19*).

We performed voluntary HEV RNA NAT of routinely collected blood donation samples retained over years and collected before 2019, when HEV screening of blood products became mandatory (*20*). Because of the retrospective nature of the analysis, donors were not excluded from donating in the interim. In addition, only a few days elapsed between each donation, resulting in dense data on viral load progression. HEV RNA–positive donors underwent serologic status follow-up, sometimes over several weeks. The data from our study provide detailed insight into the dynamics of HEV infection in asymptomatic persons as well as evaluation of viral load doubling time and half-life during the infection in this cohort.

## Material and Methods

### Blood Donors and HEV RNA screening

We retrospectively screened samples for HEV RNA, using blood collected for donation before mandatory HEV NAT screening was initiated. All donors denied having an acute illness and stated that they had no known risk factor for a viral infection.

We conducted the screening for HEV RNA in master pools of 96 samples (200 μL/donor) by using the Chemagic viral DNA/RNA reagent kit for RNA extraction (PerkinElmer Chemagen Technologies, https://www.revvity.com) and the RealStar HEV-RT-PCR Kit (Altona Diagnostic Technologies, https://altona-diagnostics.com) for amplification (95% limit of detection [LOD] 4.7 IU/mL, 95% CI 3.6 –7.6 IU/mL; 95% LOD of 451 IU/mL for a single donation in a 96-sample mini pool), as described previously (*21*). Alternatively, we extracted and amplified HEV RNA by using the AltoStar AM16 and the AltoStar HEV RNA RT-PCR Kit (Altona Diagnostic Technologies) (95% LOD 3.41 IU/mL, 95% CI 2.28–6.4 IU/mL; 95% LOD of 327 IU/mL for a single donation in a 96-sample minipool).

We quantified the viral load by using the AltoStar HEV RNA RT-PCR Kit on the Biorad CFX96 DeepWell system (Bio-Rad Laboratories, https://www.bio-rad.com). We quantified the viral load in reference to internal kit standards, calibrated against World Health Organization International Standard (PEI code 6329/10).

### Calculation of Generation Time

**Figure 3:**
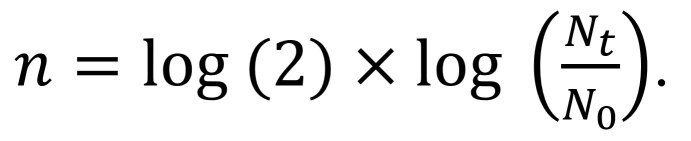
Formula. The calculation of the kinetics of the hepatitis A virus.

Assuming exponential growth, we determined the doubling times and the half-lives between 2 data points to be comparable to the calculation of the kinetics of the hepatitis A virus (*11*), based on the formula (Figure 3)

.

*N*_0_ corresponds to the viral load at the beginning of the period, *N*_t_ to the viral load after the considered time t, and *n* to the number of doublings. We used division to calculate the number of doublings per day.

For each person, we determined and graphically displayed the median of the virus doubling time and the half-life. To exclude that the parameters at the beginning and end of phases differ compared with the core region of the course, we considered 2 intervals for calculation. The first interval includes the calculations between all data points (first, last, and peak; whole course). The second interval includes only the core region (excluding first, last, and peak; trimmed course). Considering the doubling time and half-life, we appended the courses of viral loads from 7 donors, for whom other kinetic data had been previously published (*21*).

### Serologic Testing

We traced seroconversion of HEV RNA-positive blood donors, evaluating HEV IgM and IgG titers by using HEV ELISA Kits (Wantai, https://www.sanbio.nl) according to manufacturer instructions. We determined the semiquantitative evaluation of titers as the signal to cutoff ratio (S/CO). 

We visualized the data by using GraphPad Prism 9.0 software (GraphPad Software, https://www.graphpad.com). All calculations were also performed by using GraphPad Prism 9. 

## Results

We analyzed progression of HEV viremia in 32 HEV RNA-positive persons. On average, there were 4 days (interquartile range [IQR] 3–7 days) between donations. Analysis and quantification of the viral load and determination of the serostatus in all subsequent samples from the corresponding persons provided deeper insight into the course of HEV infection in terms of doubling time and clearance (half-life) of the virus, as well as seroconversion.

**Figure 1:**
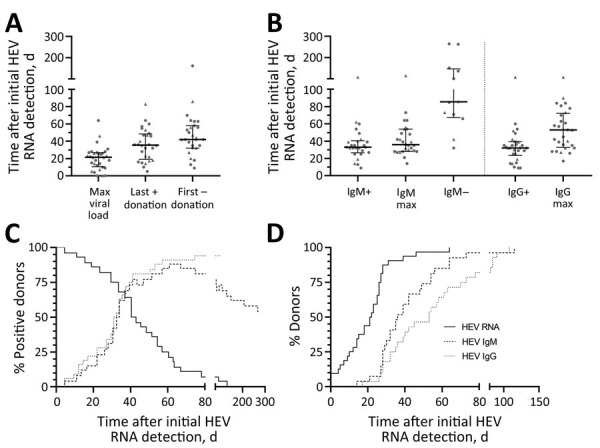
Progression of HEV infection in asymptomatic persons determined from retrospectively tested blood samples, Germany. A) Days at which the maximum viral load was reached as well as the time points of last HEV RNA–positive and the first HEV RNA–negative donation. B) Serostatus for HEV IgM and HEV IgG revealing the time points of primary HEV IgM or IgG detection, maximum ratio signal to cutoff, and loss of detectability of HEV IgM. In panels A and B, data points indicate each person; error bars indicate medians with interquartile ranges; circles indicate donors who donated HEV-negative blood before first detection of HEV RNA; and triangles indicate persons who did not donate HEV-negative blood before first detection of HEV RNA. C) percentages of persons who were positive for the markers HEV RNA, HEV IgM, and HEV IgG. D) Progression curves for the percentages of persons in whom the maximum of those markers was exceeded, depending on the time since initial HEV RNA detection. HEV, hepatitis E virus; max, maximum; +, positive; –, negative.

On average, HEV viremia reached maximum viral load after 22 days (IQR 10–27 days) (Figure 1). The highest viral load was detected at the initial positive donation for 3 persons. Note that the maximum viral load for those persons was reached before the measurement period. The longest period until detection of the maximum was 64 days. An HEV RNA–negative donation marking the end of the infection was recorded for 28 donors on day 39 (IQR 25–56 days, total range 9–154 days). The last positive donation for those donors was at a median of 36 days (IQR 19–49 days, total range 5–83 days) after the first HEV RNA–positive donation (Figure 1, panel A).

HEV IgM was detected in 28 persons on average at day 33 (IQR 25–40 days, total range 7–108 days) initially and reached the maximum S/CO at day 36 (IQR 28–54 days, total range 14–115 days). HEV IgM detectability disappeared for 13 persons at a median of 85 days (IQR 59–108 days, total range 42–265 days). HEV IgG was detected in 31 persons on average at day 32 (IQR 24–39 days, total range 9–108 days) initially and reached the maximum S/CO at day 53 (IQR 32–72 days, total range 17–108 days) (Figure 1, panel B). For 3 persons, HEV IgM could not be detected at any time. For 1 person, no seroconversion was detected because the last donation analyzed corresponded to day 26 after initial detection of HEV RNA.

Over time, decline of the proportion of RNA positive-persons and increased proportion of persons positive for HEV IgM and IgG was recorded as expected. Of note, during the rise of the curves, the proportions did not differ significantly between persons positive for HEV IgM or for HEV IgG, indicating simultaneous production onset of antibodies of both classes. However, the decline of the percentage of HEV IgM–positive persons again represents the loss of IgM titer for some (Figure 1, panel C).

When comparing the proportions of persons for whom HEV RNA viral load, HEV IgM, and IgG S/CO maximums were exceeded, we observed that maximum viral load was reached earlier than maximum HEV IgM and IgG S/CO. The proportion of persons for whom maximum HEV IgG S/CO was attained is always lower than the proportion for whom maximum HEV IgM S/CO was attained during the observed period, indicating an earlier peak in HEV IgM than IgG titers (Figure 1, panel D).

**Figure 2:**
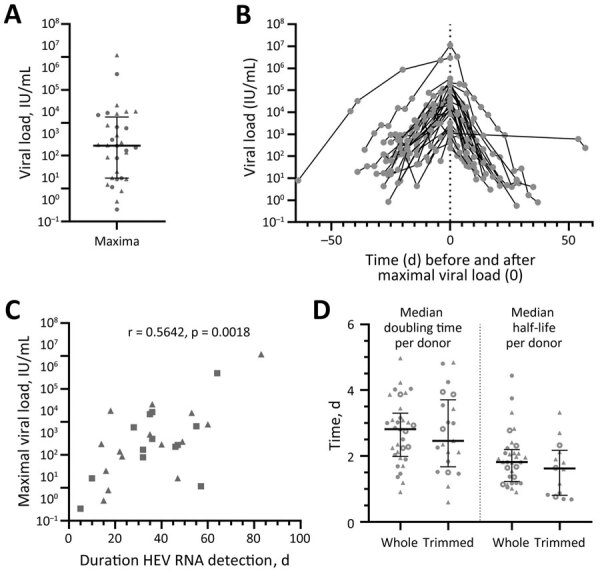
Hepatitis E virus (HEV) viral load in asymptomatic persons determined from retrospectively tested blood samples, Germany. A) Maximum viral load for each person (n = 32). B) Viral loads during the infection have been overlayed for all persons depending on the timepoint when the maximum viral load (set as day 0) was reached. C) Spearman coefficient calculated for the correlation of the maximum viral load and the duration of HEV RNA detection in the blood for persons with confirmed end of infection by HEV RNA–negative donation (n = 28). D) Doubling time was determined in the rising phase of the viral load, whereas the half-life was determined in the declining phase for the whole or a trimmed course. Data points indicate each person; error bars indicate medians with interquartile ranges; circles indicate donors who donated HEV-negative blood before first detection of HEV RNA (n = 14); and triangles indicate persons who did not donate HEV-negative blood before first detection of HEV RNA (n = 18). Data points calculated from data extracted from Vollmer et al. are displayed as open circles (n = 7) (*21*). HEV, hepatitis E virus.

In addition to determining the time course of infection markers, we quantified viral load for several follow-up samples to identify the maximum viral load and development of the viral load during infection, leading to our analysis of the correlation between the maximum viral load and duration of HEV RNA positivity as well as the calculation of the doubling time and the half-life of the viral load (Figure 2). The average maximum viral load amounted to 2.0 × 10^4^ IU/mL (IQR 2.0 × 10^3^–1.5 × 10^5^ IU/mL, total range 2.3 × 10^2^–1.1 × 10^7^ IU/mL) (Figure 2, panel A). The overlayed development of the viral load in the time course for all persons (Figure 2, panel B) demonstrates an approximated exponential increase and decrease of viral load. Based on these data, plotting of the maximum viral load against the duration of the infection showed a weak correlation, with a coefficient of r = 0.5642 at a significance level of p = 0.0018 (Figure 2, panel C).

In addition to the kinetics we observed (n = 32) and with regard only to the doubling time and half-life, we included in our calculation data collected from asymptomatic blood donors published before (n = 7), which have not yet been examined for those parameters (*21*). We differentiated between the median doubling time per person in the whole course and in the trimmed course (excluding values corresponding to the first or last HEV RNA–positive sample or the maximum viral load). The calculation in the rising phase of the viral load revealed an average doubling time of 2.8 days (IQR 2.0–3.3 days, total range 0.9–5.0 days; n = 33) during the whole course and of 2.4 days (IQR 1.7–3.7 days, total range 0.6–4.8 days; n = 21) during the trimmed course. The calculations for the declining phase of the viral load resulted in an average half-life of 1.8 days (IQR 1.2–2.2 days, total range 0.9–4.4 days; n = 32) during the whole course and of 1.6 days (IQR 0.8–2.2 days, total range 0.7–3.3 days; n = 14) during the trimmed course (Figure 2, panel D).

## Discussion

The course progression of a viral infection is usually difficult to follow because viremia often proceeds unobserved before symptom onset. For HEV, a large proportion of infections are additionally assumed to be asymptomatic and self-limiting (*22*), which is reflected in the discrepancy between the number of reported cases and the high HEV IgG seroprevalence in the general population (*23*). The introduction of standard screening of blood donors in some countries in Europe has enabled early detection of viremia (*10*). However, doing so gives rise to a second problem with analyzing viral kinetics, because affected donors are excluded from further donations for months, so no progression statistics can be applied. Symptomatic patients are also rarely suitable for analysis of viremia in the second half of infection because treatments (e.g., ribavirin) inherently affect the course progression (*24*,*25*).

Because our studied data represent asymptomatic infected persons retrospectively identified from retained samples stored over years, closely meshed follow-up samples are available for viral load, and those data provide a unique opportunity to gain understanding of kinetics in asymptomatic blood donors. Blood donations from asymptomatic donors pose a risk for development of transfusion-transmitted severe and chronic hepatitis E in immunosuppressed patients and recipients of solid organ transplants (*5*).

In the overall evaluation of our results, we mainly discuss median values that were calculated. It should be noted that infection characteristics differed greatly among individual donors. In that context, case–control studies might be appropriate for assessing differences among donors in terms of kinetics shift in context with secondary factors (e.g., patient age, virus genotype). Of note, to evaluate the effect of specific mutations on doubling time and maximum viral load possibly correlating with severity of infection, as has been shown for hepatitis B virus infections, our data provide necessary baseline information for infection courses (*14*).

Several serologic studies of kinetics during HEV infection have been published and state detection of HEV RNA in blood over 4 weeks, which is comparable to the 36 days (or 5 weeks) of viremia that we detected (*16*–*18*). Although both HEV IgM and IgG have been described as appearing ≈2 weeks after first detection of HEV RNA during acute hepatitis E, we observed a much longer period of 32 (IgM) and 33 (IgG) days, although similar simultaneous onset of both immunoglobulins was confirmed (*17*,*18*,*26*). We were able to display a fast increase in HEV IgM S/CO reflected by the reaching of maximum HEV IgM S/CO at day 36, followed by a slow decrease leading to titers below the LOD in around one third of the donors at a median of day 85. Meanwhile, HEV IgG S/CO increased more slowly but stayed detectable at a high level in all persons. Those kinetics are in line with the general expectations for the course of the serologic response during a self-limiting infection (*27*,*28*).

The data presented here on the viremic course of asymptomatic persons have been published only to a limited extent because of the restrictions mentioned above. A previous study of ours, with data from 10 asymptomatic persons, provided insight into the viremic kinetics and reported a median maximum viral load of 1.88 × 10^4^ IU/mL reached at day 23 and a total interval of HEV RNA positivity of 28 days (*21*), which is in line with the maximum viral load of 2.0 × 10^4^ IU/mL reached at day 22 and a total period of 36 days of RNA positivity that we report. Therefore, we considered it reasonable to include the data from that study when calculating the doubling time and half-life of HEV viral loads.

A comparison of data from asymptomatic persons with viremia characteristics of patients after solid organ transplantation published by Pas et al. resulted in a striking discrepancy (*29*). First, the discrepancy is reflected in the fact that, in the Pas et al. study, the median time between detection of HEV RNA and detection of HEV IgG was 124 days, whereas in our study that period was only 33 days. Second, in that study, chronic hepatitis E developed in 11 of 12 organ recipients with a median duration of 16 months, whereas, among the asymptomatic donors in our study, chronic hepatitis E developed in none (>3 months viremia) with a median infection period of 36 days. Consequently, the kinetics of acute and chronic hepatitis E need to be differentiated.

When considering the doubling time and half-life of viral load during HEV viremia, comparative data (e.g., from symptomatic patients) are missing. Our calculations resulted, depending on the analysis window considered, in a doubling time of 2.8–2.4 days and a half-life of 1.8–1.6 days. The values in the whole or the trimmed core intervals were similar, indicating uniform growth and fall of the viral load. Therefore, we did not take complex calculations for different phase into account as has been done for HBV, for example (*30*). HEV infections are closely related to HAV infections in terms of not only the route of transmission and the self-limiting character but also the signs/symptoms (*15*,*31*). A higher maximum viral load of 2.6 × 10^7^ IU/mL, a longer infection duration of 106 days, and a doubling time of 17.5 hours for asymptomatic HAV positive blood donors have been published (*11*). A comparably low doubling time of 13.8 hours has been demonstrated in HCV-infected liver transplant recipients (*13*). For HBV-infected persons, a doubling time of 2.5–3.7 days and a half-life of 1.6–3.7 days has been reported, which is more similar to the HEV kinetics (*12*,*14*). At the same time, a stable maximum viral load for HBV of 10^8^–10^9.5^ IU/mL has been detected in this context and was interpreted as the viral load associated with the infection of nearly all hepatocytes (*12*,*30*). The median maximum HEV viral load that we detected is a few logs lower and fluctuated between donors, not accounting for infection of all hepatocytes.

In conclusion, our data provide insight into the kinetics of asymptomatic HEV infection. Although key characteristics (e.g., humoral immune response, maximum viral load, and duration of viremia) confirmed results of previous studies, doubling time and half-life of the viral load were additionally determined. Comparison with the course of infection in organ transplant recipients as well as patients with other viral liver infections revealed characteristic deviations, highlighting that HEV infection in healthy persons is less extensive and leads to fewer health impairments and that risk factors such as immunosuppression influence the infection. Our data provide a baseline for evaluating self-limiting HEV infection. With the addition of secondary factors such as age or immune status, it can be determined which factors influence the kinetics of the infection and represent a risk to, for example, recipients of transfusion-transmitted hepatitis E.

## References

[1] Cao D, Meng XJ. Molecular biology and replication of hepatitis E virus. Emerg Microbes Infect. 2012;1:e17. 10.1038/emi.2012.7 26038426 PMC3630916

[2] Purdy MA, Harrison TJ, Jameel S, Meng XJ, Okamoto H, Van der Poel WHM, et al.; Ictv Report Consortium. ICTV virus taxonomy profile: Hepeviridae. J Gen Virol. 2017;98:2645–6. 10.1099/jgv.0.000940 29022866 PMC5718254

[3] Horvatits T, Varwig-Janssen D, Schulze Zur Wiesch J, Lübke R, Reucher S, Frerk S, et al. No link between male infertility and HEV genotype 3 infection. Gut. 2020;69:1150–1. 10.1136/gutjnl-2019-319027 31118248

[4] Cheung CKM, Wong SH, Law AWH, Law MF. Transfusion-transmitted hepatitis E: What we know so far? World J Gastroenterol. 2022;28:47–75. 10.3748/wjg.v28.i1.47 35125819 PMC8793017

[5] Westhölter D, Hiller J, Denzer U, Polywka S, Ayuk F, Rybczynski M, et al. HEV-positive blood donations represent a relevant infection risk for immunosuppressed recipients. J Hepatol. 2018;69:36–42. 10.1016/j.jhep.2018.02.031 29551705

[6] Harvala H, Hewitt PE, Reynolds C, Pearson C, Haywood B, Tettmar KI, et al. Hepatitis E virus in blood donors in England, 2016 to 2017: from selective to universal screening. Euro Surveill. 2019;24:1800386. 10.2807/1560-7917.ES.2019.24.10.1800386 30862338 PMC6415500

[7] Aggarwal R, Jameel S, Hepatitis E. Hepatitis E. Hepatology. 2011;54:2218–26. 10.1002/hep.24674 21932388

[8] World Health Organization (WHO). Hepatitis E [cited 2023 Jul 26]. https://www.who.int/news-room/fact-sheets/detail/hepatitis-e

[9] Pérez-Gracia MT, Suay B, Mateos-Lindemann ML. Hepatitis E: an emerging disease. Infect Genet Evol. 2014;22:40–59. 10.1016/j.meegid.2014.01.002 24434240

[10] Federal Ministry of Justice. Announcement on the authorisation of medicinal products—defence against drug risks - order to test blood donors to prevent the transmission of hepatitis E virus through blood components for transfusion and stem cell preparations for haematopoietic reconstitution [in German] [cited 2023 Jul 26]. https://www.bundesanzeiger.de/pub/publication/8ziFMqlkUHaYCwHxuin?0

[11] Schoch S, Wälti M, Schemmerer M, Alexander R, Keiner B, Kralicek C, et al. Hepatitis A virus incidence rates and biomarker dynamics for plasma donors, United States. Emerg Infect Dis. 2021;27:2718–824. 10.3201/eid2711.204642 34670659 PMC8544996

[12] Whalley SA, Murray JM, Brown D, Webster GJM, Emery VC, Dusheiko GM, et al. Kinetics of acute hepatitis B virus infection in humans. J Exp Med. 2001;193:847–54. 10.1084/jem.193.7.847 11283157 PMC2193367

[13] Garcia-Retortillo M, Forns X, Feliu A, Moitinho E, Costa J, Navasa M, et al. Hepatitis C virus kinetics during and immediately after liver transplantation. Hepatology. 2002;35:680–7. 10.1053/jhep.2002.31773 11870384

[14] Yoshikawa A, Gotanda Y, Itabashi M, Minegishi K, Kanemitsu K, Nishioka K. Hepatitis B NAT virus-positive blood donors in the early and late stages of HBV infection: analyses of the window period and kinetics of HBV DNA. Vox Sang. 2005;88:77–86. 10.1111/j.1423-0410.2005.00602.x 15720604

[15] Velavan TP, Pallerla SR, Johne R, Todt D, Steinmann E, Schemmerer M, et al. Hepatitis E: An update on One Health and clinical medicine. Liver Int. 2021;41:1462–73. 10.1111/liv.14912 33960603

[16] Huang S, Zhang X, Jiang H, Yan Q, Ai X, Wang Y, et al. Profile of acute infectious markers in sporadic hepatitis E. PLoS One. 2010;5:e13560. 10.1371/journal.pone.0013560 21042408 PMC2958841

[17] Zhang J, Ge SX, Huang GY, Li SW, He ZQ, Wang YB, et al. Evaluation of antibody-based and nucleic acid-based assays for diagnosis of hepatitis E virus infection in a rhesus monkey model. J Med Virol. 2003;71:518–26. 10.1002/jmv.10523 14556264

[18] Clayson ET, Myint KSA, Snitbhan R, Vaughn DW, Innis BL, Chan L, et al. Viremia, fecal shedding, and IgM and IgG responses in patients with hepatitis E. J Infect Dis. 1995;172:927–33. 10.1093/infdis/172.4.927 7561211

[19] Hoofnagle JH, Nelson KE, Purcell RH, Hepatitis E. Hepatitis E. N Engl J Med. 2012;367:1237–44. 10.1056/NEJMra1204512 23013075

[20] Vollmer T, Diekmann J, Johne R, Eberhardt M, Knabbe C, Dreier J. Novel approach for detection of hepatitis E virus infection in German blood donors. J Clin Microbiol. 2012;50:2708–13. 10.1128/JCM.01119-12 22675127 PMC3421526

[21] Vollmer T, Diekmann J, Eberhardt M, Knabbe C, Dreier J. Hepatitis E in blood donors: investigation of the natural course of asymptomatic infection, Germany, 2011. Euro Surveill. 2016;21:30332. 10.2807/1560-7917.ES.2016.21.35.30332 27608433 PMC5015460

[22] Pischke S, Behrendt P, Bock CT, Jilg W, Manns MP, Wedemeyer H. Hepatitis E in Germany—an under-reported infectious disease. Dtsch Arztebl Int. 2014;111:577–83. 25249359 10.3238/arztebl.2014.0577 PMC4174681

[23] Faber M, Willrich N, Schemmerer M, Rauh C, Kuhnert R, Stark K, et al. Hepatitis E virus seroprevalence, seroincidence and seroreversion in the German adult population. J Viral Hepat. 2018;25:752–8. 10.1111/jvh.12868 29377436

[24] Ambrosioni J, Mamin A, Hadengue A, Bernimoulin M, Samii K, Landelle C, et al. Long-term hepatitis E viral load kinetics in an immunocompromised patient treated with ribavirin. Clin Microbiol Infect. 2014;20:O718–20. 10.1111/1469-0691.12576 24476456

[25] Lhomme S, Kamar N, Nicot F, Ducos J, Bismuth M, Garrigue V, et al. Mutation in the hepatitis E virus polymerase and outcome of ribavirin therapy. Antimicrob Agents Chemother. 2015;60:1608–14. 10.1128/AAC.02496-15 26711757 PMC4775994

[26] Hofmann AF. Chemistry and enterohepatic circulation of bile acids. Hepatology. 1984;4(Suppl):4S–14S. 10.1002/hep.1840040803 6384004

[27] Miller JFAP. Cellular basis of the immune response. Acta Endocrinol Suppl (Copenh). 1975;194(Supplement):55–76. 47689

[28] Perelson AS, Goldstein B, Rocklin S. Optimal strategies in immunology III. The IgM-IgG switch. J Math Biol. 1980;10:209–56. 10.1007/BF00276984 7252371

[29] Pas SD, de Man RA, Mulders C, Balk AHMM, van Hal PTW, Weimar W, et al. Hepatitis E virus infection among solid organ transplant recipients, the Netherlands. Emerg Infect Dis. 2012;18:869–72. 10.3201/eid1805.111712 22516170 PMC3358074

[30] Tsiang M, Rooney JF, Toole JJ, Gibbs CS. Biphasic clearance kinetics of hepatitis B virus from patients during adefovir dipivoxil therapy. Hepatology. 1999;29:1863–9. 10.1002/hep.510290626 10347131

[31] Naoumov NV. Hepatitis A and E. Medicine (Baltimore). 2007;35:35–8. 10.1053/j.mpmed.2006.10.004

